# Collaborative Robotics, Mobile Platforms, and Total Laboratory Automation in Clinical Diagnostics

**DOI:** 10.3390/diagnostics16040518

**Published:** 2026-02-09

**Authors:** Shuvam Mukherjee, Charlie Lambert, Yizhi Zhou, Steven Kan, Jianfei Yang, Guochun Liao, Steven Flygare, Robert S. Ohgami

**Affiliations:** 1 Department of Computer Engineering, University of Maryland, College Park, MD 20742, USA; shuvam1024@gmail.com; 2 Robotics Program, College of Engineering, University of Utah, Salt Lake City, UT 84108, USA; charlielambert@gmail.com; 3 Department of Electrical and Computer Engineering, George Mason University, Fairfax, VA 22030, USA; yzhou26@gmu.edu; 4 Robotic Assistant Inc., Salt Lake City, UT 84108, USA; steven.x.kan@rassistant.ai (S.K.); guochun.liao@rassistant.ai (G.L.); 5 School of Mechanical and Aerospace Engineering, Nanyang Technological University, Singapore 639798, Singapore; jianfei.yang@ntu.edu.sg; 6 Fabric Genomics, Salt Lake City, UT 84108, USA; stevendflygare@rassistant.ai; 7 Department of Pathology, University of Utah, Salt Lake City, UT 84108, USA; 8 Institute for Research and Innovation in Diagnostic and Precision Medicine, ARUP Laboratories, Salt Lake City, UT 84108, USA

**Keywords:** clinical laboratory automation, total laboratory automation, collaborative robots, autonomous mobile robots, humanoid robot, CLIA, FDA, pathology, IVD regulation

## Abstract

Clinical diagnostic laboratories continue to face growing pressure from rising test volumes, increasingly complex testing menus, significant workforce shortages, and expectations for faster turnaround times at sustainable cost. Total laboratory automation (TLA) has become a central strategy for improving efficiency in high-volume laboratories, where integrated systems from Abbott, Roche, Siemens Healthineers, and Beckman Coulter have demonstrated substantial reductions in turnaround time, error rates, and labor requirements. Evidence across multiple health systems shows that TLA improves performance and stabilizes laboratory operations even during workload peaks. Despite these gains, large segments of pre-analytical and post-analytical workflows remain manual, especially tasks related to specimen transportation, bench-level manipulation, instrument tending, and troubleshooting. Recent progress in collaborative robotics (cobots), autonomous mobile robots (AMRs), and hospital service robots demonstrates that these technologies can complement TLA by addressing not only the logistical and dexterous tasks that fixed automation lines cannot reach but also enabling robots that can work safely right alongside humans in a shared space. Cobots have shown sub-millimeter precision in colony picking and other fine-motor tasks, though typically at lower throughputs than dedicated track modules, and AMRs have demonstrated reliable transport of pathology carts and medical supplies through large clinical environments. Meanwhile, humanoid-capable mobile manipulators, like Moxi from Diligent Robotics, deployed in hospitals are already completing hundreds of thousands of supply deliveries, indicating real-world significance. Here, we integrate technical, regulatory, operational, and business perspectives on TLA, collaborative robotics, and mobile platforms. We discuss real-world efficiency gains, regulatory expectations under the CLIA and United States FDA, and the emerging case for hybrid automation ecosystems that combine TLA islands, cobotic workcells, AMRs, and AI-enabled orchestration. We argue that the next decade of laboratory automation will move beyond monolithic tracks with robots toward flexible, modular robotic systems designed to operate safely together with humans and to augment the increasingly strained laboratory workforce. This not only allows clinical staff to dedicate more time to patient care but also ensures greater reliability and scalability for essential services throughout demanding hospital environments.

## 1. Introduction

Clinical laboratories are experiencing simultaneous increases in testing volume, diagnostic complexity, and expectations for rapid and accurate reporting. These demands are unfolding against a backdrop of chronic shortages of qualified medical technologists and pathologists, a problem described across multiple regions and expected to worsen as experienced personnel retire faster than they can be replaced, forcing the industry to adapt through innovation [1,2]. At the same time, the global IVD market is projected to reach more than $113 billion USD in 2026, with molecular diagnostics, digital pathology, and automation representing central drivers of growth [3].

TLA has emerged as a response with the necessary high-precision and high-throughput automation that would drive the growth of the multi-billion-dollar IVD market. Since the early 2000s, vendors such as Abbott, Roche, Beckman Coulter, and Siemens Healthineers have developed integrated automation lines capable of automating pre-analytical, analytical, and post-analytical tasks. TLA has evolved from basic conveyor tracks to highly instrumented systems that manage sample receipt, sorting, centrifugation, decapping, aliquoting, analytical routing, archival storage, and retrieval [4].

Yet, complete end-to-end automation remains elusive. Even in advanced TLA installations, humans continue to perform tasks such as transporting carts, loading and unloading instruments, handling irregular specimen types, solving exceptions, and performing manual tasks that lie outside the fixed geometry of automation tracks. Moreover, workforce studies show that while TLA reduces repetitive tasks, it also increases the need for personnel skilled in troubleshooting, IT/programming, middleware management, and quality oversight [1,5].

At the same time, robotics has experienced rapid expansion in hospitals and research laboratories outside traditional clinical laboratories. Collaborative robots (cobots) now perform precision bench tasks, AMRs traverse corridors and elevators autonomously, and humanoid-capable mobile manipulators shoulder supply logistics in hospitals [6,7]. Robotic-assisted surgery (e.g., the da Vinci system) illustrates the broader adoption of robotics in hospital settings, though it is primarily tele-operated and is not directly comparable to autonomous, high-throughput batch processing in clinical laboratories.

Given these developments, the next era of clinical laboratory automation may be defined not by a single all-encompassing track but by a hybrid, modular ecosystem that integrates TLA islands, robotic workcells, AMRs, and orchestration software capable of safely coordinating human–robot workflows within established CLIA and FDA regulatory frameworks.

Here, we evaluate how TLA, cobots, and AMRs can be combined within CLIA and FDA frameworks to strengthen laboratory resilience, address workforce shortages, and expand automation into pre- and post-analytical domains.

## 2. Search Methodology

A review of the peer-reviewed literature, regulatory documents, and selected industry publications was performed, focusing on laboratory automation, collaborative robotics, mobile robotic platforms, and their intersection with clinical workflows.

We searched PubMed, Web of Science, and Google Scholar for articles published between 2010 and December 2025 using combinations of the terms “total laboratory automation,” “clinical laboratory automation,” “laboratory robotics,” “collaborative robot,” “cobot,” “autonomous mobile robot,” “service robot,” “microbiology automation,” “CLIA,” “IVD regulation,” and “workflow automation,” together with “laboratory,” “pathology,” or “clinical diagnostics”.

We prioritized empirical studies reporting objective outcomes such as TAT (Turn Around Time), error rates, labor metrics, and productivity, as well as authoritative reviews, consensus statements, and regulatory publications from the Centers for Medicare & Medicaid Services (CMS) and the United States FDA.

Industry case studies from Siemens Healthineers, Roche Diagnostics, Beckman Coulter, Abbott, Diligent Robotics, and Relay Robotics were included when data were specific and verifiable and when they addressed operational considerations relevant to clinical laboratories. Our objective was to identify representative findings and synthesize emerging patterns relevant to clinical laboratories planning near-term automation strategies.

In addition to traditional academic and laboratory medicine sources, we also included research from industrial engineering, hospital logistics, and human–robot interaction to capture emerging evidence on mobile manipulation and intralogistics. We also incorporated work from nursing and service-robotics domains that systematically classify the risks and mitigation strategies required for safe integration of mobile robotic assistants in hospitals, which are directly relevant to clinical laboratory deployment. This broader perspective was necessary to understand how robotics proven in adjacent hospital environments can be extended into pre-analytical and post-analytical laboratory workflows.

## 3. State of the Art in Laboratory Automation

### 3.1. Current State of Core Laboratory Automation

Core laboratory TLA implementations consistently show measurable gains in efficiency, reproducibility, and cost control (Table 1). Early work describing the origins of TLA emphasized that success hinges on redesigning workflow architecture rather than bolting automation onto existing processes [4]. This principle has been reinforced by subsequent real-world installations. For instance, in Italy, a major laboratory consolidation project demonstrated that moving multiple labs into a single TLA-enabled core improved sample management, shortened internal transport times, and stabilized TAT under heavy load [8]. In a separate study focused on cardiac troponin I testing, the introduction of TLA not only reduced median TAT but also decreased variability by minimizing outliers, which in turn reduced inappropriate STAT ordering [9].

Additional evaluations have highlighted the importance of pairing TLA with advanced pre- and post-analytic modules, such as automated centrifugation, decapping/recapping, cell sorting, aliquoting, or sample quality control. Integrating refrigerated storage and middleware has produced substantial gains in operational efficiency in some labs [13]. Expanding automation to include test consolidation significantly reduced tube touches and labor requirements while maintaining analytic quality in others [14]. Broader key-performance analysis studies have demonstrated the same pattern: once a TLA system is properly integrated, laboratories experience reduced manual workload and more predictable, controllable operations [15]. Many experts emphasize that these benefits are real but dependent on thoughtful alignment between platform capabilities and the specific laboratory context, as well as the workflow [16].

Economic data strengthen this interpretation. A formal cost-effectiveness study in a high-volume chemistry and immunoassay laboratory showed a 37% reduction in manual processing steps, shorter median TAT for core analytes, and an incremental cost-effectiveness ratio supportive of a four to five-year payback period [17]. A longitudinal analysis from a major academic center revealed that the incremental adoption of robotics and advanced informatics drove an almost tenfold gain in productivity. During this period, the inflation-adjusted cost per test declined from $0.79 to $0.15, and the laboratory successfully maintained stable STAT turnaround times even while facing a significant increase in STAT demand [18].

These findings align with broader implementation reviews indicating that maximum performance gains in laboratories are achieved by combining TLA with lean methods, active staff engagement, and iterative redesign of steps like specimen routing, rather than merely replacing manual steps with machines [19]. Institutional experience from a tertiary-care hospital in Korea similarly has demonstrated that transitioning from subtotal automation to full TLA reduced manual labor, shortened TAT, and achieved a payback period of under five years based on labor savings alone [17]. Additionally, longitudinal experience in New York confirms a parallel trend across multiple decades of automation [18]. Viewed in the wider healthcare context, these improvements occur in a sector that constitutes a small fraction of total healthcare spending yet informs most inpatient clinical decisions. At the same time, contemporary error-tracking studies show that 50–70% of all detectable laboratory errors still arise in the pre-analytical phase, underscoring that even advanced TLA requires targeted interventions in ordering, collection, and specimen handling to capture the full benefit of automation [19,20,21].

### 3.2. Automation in Clinical Microbiology

Microbiology automation has accelerated over the past decade with the introduction of automated streakers, intelligent incubators, high-resolution imaging systems, and AI-supported plate-reading platforms [22]. Early work has shown that automated microbiology lines can shorten reporting times for urine cultures, although the gains have varied depending on reading schedules and staffing patterns [23]. Pasqualetti et al. demonstrated reductions in delays for add-on testing and noted improved process stability once routine steps were automated [24]. Despite these advances, microbiology remains highly interpretive and labor-intensive. Colony picking, Gram stain preparation, review of complex polymicrobial specimens, and confirmatory testing still fall outside the capabilities of most TLA systems, which has motivated the exploration of collaborative robotic arms to close these gaps.

Larger datasets have quantified the combined impact of TLA and deliberate workflow redesign. Zimmermann et al. reported across four laboratories of different sizes that integrating automated streaking, incubation, and imaging systems significantly reduced hands-on time and enabled earlier availability of negative results, with economic benefit driven largely by FTE redeployment to other tasks [25]. In parallel, AI-powered automated microscopy platforms have demonstrated advantages in disease detection for malaria, tuberculosis, and sickle cell disease, particularly in low-resource settings such as Africa, as exemplified by systems developed by companies including Cephla Inc., Mountain View, CA, USA. In an academic regional hospital, Gonzalez-Ortiz et al. further showed that a sequential implementation strategy—first standardizing culture workflows, followed by the introduction of TLA—shortened identification turnaround times for urine, blood, and wound cultures by up to 19 h and increased the proportion of results released within targeted time windows [26]. Additional reviews and cohort studies indicate that these gains are most consistently reproduced when automation is coupled with rationalized culture order sets and clear rules for which specimen types remain on manual pathways [27,28]. Collectively, these data suggest that microbiology can achieve performance improvements comparable to chemistry and hematology, but only if automation is implemented as part of a broader workflow redesign that includes accessioning, incubation, and reading practices.

### 3.3. Workforce and Organizational Impact

Workforce studies have shown that automation alters job content without eliminating the need for skilled personnel. In a recent study, Al Naam et al. showed that TLA allowed their tertiary hospital to absorb increased test volumes without increasing staff, but also shifted job roles toward oversight and troubleshooting. Overall, automation has been shown to improve productivity but additionally requires new technical skills and more frequent interaction with middleware and IT systems [2]. Indeed, Genzen et al. have argued that successful automation depends on investment in not only robotics technology but also informatics skills and robust governance structures [5].

As automation increases, so does the need for technologists trained in exception handling, system monitoring, middleware logic, and robotic interaction. This aligns with broader trends in digital pathology and molecular diagnostics, where constant improvement in informatics skills is increasingly central.

Contemporary quality studies have further clarified how automation reshapes the labor profile in clinical laboratories. In one example, interventions in a large university hospital, combining standardization, staff education, and targeted process changes, reduced pre-analytical error rates from 0.42% to 0.32%, with the greatest improvements seen in specimen rejection and misidentification categories [29]. Several reviews of pre-analytical performance emphasize that 60–70% of detectable laboratory errors still occur before analysis—specifically during test ordering, phlebotomy, labeling, and transport. These studies argue that while automation and digital tracking are essential, they must be supported by robust governance structures and comprehensive staff training to be truly effective [21]. From a workforce perspective, this means that technologists should increasingly act as supervisors of complex automated flows, designers of quality indicators, and investigators of rare but high-impact failures rather than as manual processors of specimens. Strategic workforce planning, therefore, needs to incorporate both advanced technical skills and structured pathways for upskilling existing staff into automation, cutting-edge software, and informatics, as well as robotics knowledge [19].

### 3.4. Collaborative Robotic Arms in Laboratory Settings

Collaborative robots are designed to operate safely alongside human workers. In the first detailed study of a cobot in clinical microbiology, Baumkircher et al. detailed the results of a cobot performing colony picking using kinesthetic motion teaching and dynamic movement primitives [30]. Their cobot demonstrated sub-millimeter precision and MALDI-TOF (Matrix-Assisted Laser Desorption/Ionization Time-of-Flight Mass Spectrometry) identification performance comparable to skilled technologists. Cobots offer a unique combination of dexterity, reprogrammability, and a compact footprint. However, cobot arms generally trade speed for flexibility; dedicated track-based TLA gantries typically provide higher throughput for standardized, repetitive tasks. Although peer-reviewed studies are still limited, emerging use cases include instrument loading, slide and cassette handling, LC-MS plate feeding, aliquoting, and sample preparation tasks that are not easily automated through fixed-function liquid handlers. Additionally, the ability to reprogram cobots quickly as workflows evolve is increasingly relevant for laboratories adopting new molecular panels or AI-enabled digital pathology workflows, where artificial intelligence is at the center to guide case triage, prioritize samples, and dynamically adjust processing steps based on image analysis or molecular results. In these settings, AI-driven decision support can be coupled with robotic execution, enabling more adaptive, demand-driven laboratory operations that reduce manual intervention, improve turnaround times, and support scalable implementation of advanced diagnostics.

Real-world deployments of cobots in hospital laboratories are beginning to illustrate how these systems complement fixed automation. For instance, at Karolinska University Hospital, an ABB collaborative robot was introduced to handle high-volume sampling tasks, taking over repetitive tube handling and freeing technologists for complex problem-solving; this resulted in improved ergonomics, more predictable throughput, and positive staff acceptance when safety features and visual cues were clearly implemented [31]. A separate installation at Gentofte Hospital in Denmark used two UR5 cobots to load and sort blood samples, enabling the laboratory to maintain a target of delivering more than 90% of results within one hour despite a 20% increase in incoming samples [32]. Outside healthcare, laboratory-focused cobot vendors highlight features such as force-limited joints, auto-centering grippers, and “free-mode” teaching that reduce engineering overhead and support rapid reconfiguration of sample-handling tasks [33]. These examples suggest that cobots are best positioned as adaptable interfaces between humans, TLA tracks, and specialized instruments, particularly where physical layouts or assay menus change frequently.

### 3.5. Autonomous Mobile Robots and Hospital Service Robots

AMRs can address logistical tasks that TLA does not solve. Published evidence for AMRs in clinical laboratories remains relatively sparse and is frequently based on pilots or vendor-led case studies; laboratories should therefore interpret reported gains cautiously and validate performance locally. In one recent study, Lewis et al. documented a Mayo Clinic pilot in which a Proxie AMR transported specimens autonomously between intake and sorting areas [34]. Travel and dwell times were predictable, and the AMR successfully completed all tasks without human escort.

Hospital service robots such as Moxi, developed by Diligent Robotics, can operate in unstructured environments, carrying medications, samples, PPE, and supplies. As of 2025, Moxi has reportedly completed more than 1.25 million deliveries across more than 25 hospitals, demonstrating the ability of service robots to operate effectively in unstructured environments while carrying medications, samples, PPE, and supplies [6,7]. Relay robots developed by Relay Robotics (formerly Savioke) extend the efficiency of automated testing by autonomously bridging the “last-mile” transport gap between patient floors and the laboratory. By integrating with elevator systems to securely deliver specimens and supplies across multi-floor facilities, these robots allow clinical staff to remain focused on patient care rather than manual logistics [35].

Current reports from research laboratories, including work from Professor Andrew Cooper’s group at the University of Liverpool and Oak Ridge National Laboratory (ORNL), describe autonomous mobile robots (AMRs) acting as physical connectors between independent analytical instruments. These examples largely originate in research chemistry rather than regulated clinical diagnostics; translating them into clinical laboratory workflows requires additional validation, documentation, and quality controls under CLIA/ISO 15189, and should not be conflated without this regulatory context. Similar architectures are emerging in clinical laboratory automation as alternatives to rigid, conveyor-based total laboratory automation. In this “hybrid ecosystem” model, AMRs link islands of automation and operate under software layers integrated with LIMS and AI-driven workflow orchestration, enabling dynamic task scheduling, priority-based sample routing, and rapid reconfiguration as test volumes and assay menus evolve. This modular approach also supports incremental validation and controlled change management, aligning with CLIA and IVDR expectations while enabling scalable adoption of advanced diagnostics [36]. This architecture closely resembles the hybrid ecosystem emerging in clinical laboratories.

Experience from broader hospital logistics provides a blueprint for integrating AMRs into diagnostic workflows. Multiple case studies of sterile-instrument logistics show that AMRs can replace trolley-based manual transport, increasing flexibility and productivity while maintaining or improving service quality and cost metrics [37]. Earlier work on hospital service robots demonstrated that autonomous vehicles moving blood samples between wards and laboratories reduced transport times from hours to minutes without requiring structural modifications to hallways or elevators [38]. More recent analyses classify in detail the operational and safety risks associated with mobile robotic assistance systems and propose mitigation strategies around fleet management, shared-space navigation, and interaction with clinical staff [39]. Vendors such as Mobile Industrial Robots (MiR) now document deployments where AMRs routinely deliver medications, linens, and supplies, freeing nursing and support staff for direct patient care and providing granular telemetry that can be repurposed as chain-of-custody evidence for specimens [40]. Together, these data support the feasibility of AMRs as a mature technology that can extend laboratory automation beyond the physical footprint of the TLA track.

### 3.6. Regulatory and Quality Frameworks

Automation in the clinical laboratory must align with existing regulatory frameworks (Table 2). Under the Clinical Laboratory Improvement Amendments (CLIA), laboratories must validate all processes that influence specimen integrity, analytical results, or reporting accuracy, including pre-analytical robotic handling [41]. Subpart K emphasizes the need for documented procedures, systematic monitoring of error rates, and prompt corrective action. For laboratories operating under international accreditation, ISO 15189 similarly requires equipment verification, ongoing performance monitoring, and documentation for automated systems, making it directly applicable to cobots and mobile robotics integrated into pre- and post-analytical workflows [42].

The FDA regulates in vitro diagnostic (IVD) devices, including their automation components. As an example, Abbott’s ACCELERATOR APS and GLP Systems Track are cleared automation platforms under 510(k), with specified intended uses related to pre-analytical sample processing [43,44]. Consequently, laboratories must ensure that robotic workflows strictly adhere to device labeling and must perform rigorous local verification studies. Furthermore, accreditation bodies such as the CAP further scrutinize robotic and automated processes during inspections, evaluating LIS interface performance, middleware rules, quality control (QC) monitoring, validation documentation, and end-to-end sample traceability.

Collaborative robots and AMRs that manipulate or transport specimens fall under CLIA quality requirements. Laboratories must document risk assessments, failure modes, and performance monitoring, and must ensure that robotic behaviors preserve chain-of-custody and environmental control (e.g., temperature, time-in-transit).

Recent studies on diagnostic errors underscore that most failures originate from pre-analytical and process issues rather than deficiencies in the analytical performance of FDA-cleared assays, which has implications for how regulators and laboratories prioritize controls. Lippi et al. describe how artificial intelligence and automation are increasingly applied to test ordering, specimen transport, and sample quality evaluation, arguing that these tools must be integrated into quality systems in a way that preserves traceability and human oversight [45]. Indeed, tightening pre-market evaluation of assays alone is unlikely to substantially reduce laboratory errors, and instead, a focus on post-market surveillance, process controls, and informatics infrastructure that detect and mitigate system-level failures is best [46]. For laboratories deploying robotics, this perspective reinforces the need to map automated steps explicitly onto CLIA quality-system elements, validate not only analytical comparability but also process reliability, and document risk assessments that specifically address human–robot interaction and mobile system behavior (Table 3). Collectively, these requirements favor modular automation approaches that support incremental validation and controlled deployment of AI-enabled workflow orchestration in clinical diagnostics.

## 4. Discussion

Here, we have shown that TLA is highly effective for laboratories with high test volumes and standardized workflows. Studies show consistent reductions in TAT, improved reliability, and decreased labor intensity when TLA is implemented thoughtfully and aligned with laboratory needs [4,9,13,14]. However, full automation remains elusive because laboratories are inherently heterogeneous environments.

Clinical impact remains an important evidence gap. Most published evaluations of TLA and robotics focus on operational endpoints (turnaround time, hands-on time, error rates, staffing) and only indirectly infer downstream benefits to patient care. Where patient-facing outcomes are reported (e.g., earlier clinical decision-making enabled by faster reporting), results are often site- and workflow-dependent. Across studies, the magnitude of benefit varies with baseline processes, test mix, and degree of workflow redesign, and some reports emphasize that automation can create new failure modes such as system-wide downtime, bottlenecks at interfaces, or large-scale propagation of pre-analytic errors if controls are inadequate [47,48].

Therefore, cobots and AMRs can fill important gaps. Cobots can automate dexterous bench tasks that fixed tracks cannot reach, while AMRs handle complex logistics across floors and buildings. Hospital deployments of Moxi and Relay robots show that mobile manipulation is already robust enough for daily operational use [6,35].

**Figure 1:**
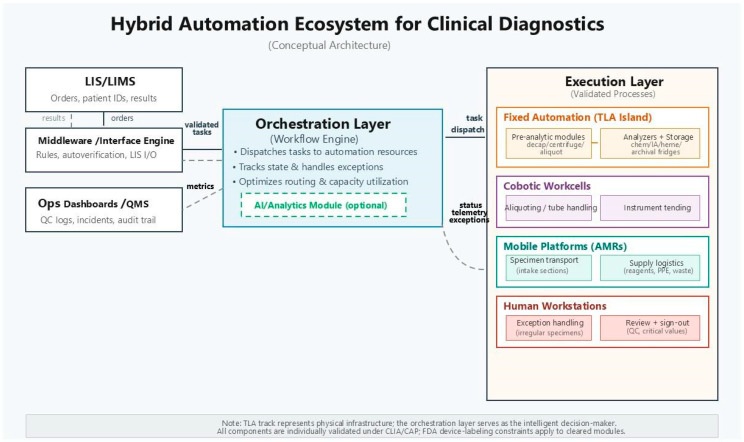
System Architecture Diagram for a hybrid automated lab with TLA. Figure Legends: LIS acts as the primary data manager and communicator for the clinical laboratory with patient information and the eventual results. The Orchestration Layer directs the samples to the appropriate analytical instrument on the TLA. The cobots (assigned for various tasks like aliquoting or LC-MS plate loading) prepare the sample for insertion into the TLA-connected instruments. TLA Track ensures that samples are efficiently and continuously delivered to the table high-throughput analyzers for the core testing phases.

Several recent studies propose that laboratories move from viewing automation as isolated chemistry or microbiology “projects” to treating them as cross-functional digital transformation. A comprehensive review of clinical microbiology automation outlines how specimen conveyance, incubation, imaging, and result reporting can be conceptualized as a continuous value stream, with hardware and software elements selected for interoperability and scalability rather than for stand-alone performance [49]. Furthermore, industry experts highlight that combining robotics with digital tracking and analytics allows laboratories to identify hidden bottlenecks, simulate “what-if” scenarios, and utilize granular, time-stamped data for advanced capacity planning and predictive maintenance [50]. In this framing, cobots, AMRs, and TLA become modular building blocks within a larger cyber–physical system rather than disconnected point solutions. We believe the next generation of laboratory automation will adopt a hybrid architecture: TLA for predictable, high-volume workflows; cobot workcells for variable bench-level tasks; and AMRs for logistical flow. As illustrated in Figure 1, these elements are unified by an orchestration layer that integrates LIS data, robotic actions, and human workflows. This framing is consistent with systems engineering and Lean Six Sigma methods used to standardize processes, identify waste, and sustain performance improvements in clinical laboratories [47].

**Figure 2:**
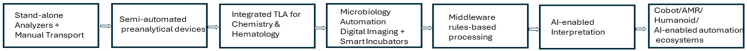
Evolution of laboratory automation.

This modular structure aligns well with CLIA, FDA, and CAP expectations, as each individual component can be validated individually while contributing to a cohesive, integrated system (Figure 2).

Health-system economics also favor this shift: laboratories represent a small fraction of total costs yet drive most diagnostic decisions, so modest gains in accuracy or turnaround time produce meaningful downstream benefits [20]. As collaborative robots and AMRs become cheaper and easier to integrate, similar returns are likely for hybrid systems that extend automation into pre-analytical and post-analytical workflows. Implemented thoughtfully, these technologies can help laboratories close workforce gaps, improve operational resilience, and strengthen patient care.

The future of laboratory automation is moving from simply connecting instruments with a track or front-end module toward more modular ecosystems in which instruments, middleware, and robotics are coordinated through an automation management layer. However, interoperability is a major practical barrier: integrating heterogeneous robots (e.g., AMRs) with proprietary analyzers and a laboratory information system (LIS) often requires custom interfaces, site-specific validation, and ongoing maintenance, which can limit scalability and reproducibility across sites.

Accordingly, the “orchestration layer” shown in Figure 1 should be viewed as an aspirational architecture rather than a fully realized off-the-shelf capability. In research automation, standards such as SiLA (and SiLA 2) provide device control and data interface conventions, but clinical laboratory devices and LIS ecosystems frequently rely on vendor-specific protocols and limited driver availability, creating an “interoperability gap” that laboratories must bridge through middleware, integration vendors, and rigorous interface verification testing [51].

AI has *potential* to improve automation performance—e.g., through predictive maintenance, anomaly detection in quality control (QC) streams, and decision support for workload balancing—but most use cases remain at an early stage and require careful prospective validation, continuous performance monitoring, and clear human oversight. In addition, operational adoption can be constrained by clinician/technologist trust and “algorithm aversion” after observing errors, emphasizing the need for transparent failure modes, escalation pathways, and training [52].

From a regulatory perspective, AI-enabled functions may fall under different oversight pathways depending on intended use (e.g., clinical decision support versus device software functions). Recent FDA guidance clarifies the scope of oversight for clinical decision support software, and complementary FDA guidance on Predetermined Change Control Plans (PCCPs) describes how certain AI-enabled device functions may be updated while maintaining a reasonable assurance of safety and effectiveness [53,54]. In Europe, guidance from regulators discusses how the EU AI Act interacts with the MDR/IVDR framework, with additional obligations that may affect both manufacturers and deployers (e.g., laboratories) of high-risk AI systems [55].

Importantly, broader automation can introduce new patient-safety risks if failures propagate at scale (e.g., specimen misrouting, systematic pre-analytic errors, or silent software/interface drift). While operational gains are well documented, direct evidence linking robotics/TLA adoption to patient outcomes remains limited; future work should quantify downstream clinical impact (e.g., time-to-treatment and diagnostic error reduction) alongside operational metrics, and should report negative findings and implementation failures to help laboratories set realistic expectations.

## Figures and Tables

**Table 1:** Representative total laboratory automation (TLA) platforms and selected operational metrics (case-study metrics often vendor-reported; interpret with context and local validation).

Vendor/System	Segment	Selected Capabilities	Representative Operational Metrics (Peer-Reviewed Where Available; Vendor-Reported Otherwise)
Siemens Aptio Automation + Atellica Solutions (Siemens Healthineers AG, Erlangen, Germany) [10,11]	Core laboratory TLA	End-to-end automation of pre-/post-analytical workflow; integrates with high-throughput analyzers; multi-discipline connectivity	DaVitaLabs: consolidated 2 labs into 1; processes up to 200,000 samples/day; >99% of tests meet TAT goals; ~10% cost per test reduction; $7.5M annual savings
Roche cobas 8100 Workflow Series (Roche Diagnostics International AG, Rotkreuz, Switzerland) [12]	Core laboratory TLA	Multi-level, bi-directional transport; automated centrifugation, decapping, and aliquoting; robust error handling	Up to 1100 samples/hour throughput; predictable TAT with STAT prioritization; hundreds of installations worldwide.
Beckman Coulter DxA 5000 (Beckman Coulter, Inc., Brea, CA, USA)	Front-end automation	Intelligent tube routing; automated sample quality checks; reduces pre-analytical variability	Standardizes TAT across routine & urgent workflows; reduction in pre-analytical errors (vendor data)
Abbott GLP Systems Track/ACCELERATOR APS (Abbott Diagnostics, Abbott Park, IL, USA)	Pre-/post-analytical automation	Modular track connecting multiple analyzers; automates loading, transport, storage, and retrieval	FDA 510(k) cleared; β-hCG study shows equivalent performance for manual vs. automated loading; designed for scalable multi-instrument connectivity.

**Table 2:** Regulatory and quality actors relevant to automation and robotics in U.S. clinical laboratories.

Actor	Primary Authority	Relevance to Automation & Robotics
CMS (CLIA Program)	Enforces 42 CFR Part 493; regulates all clinical labs	Requires validation of all automated processes; oversight of pre-analytic, analytic, and post-analytic quality; requires QC, IQCP, and risk assessments
FDA (CDRH)	Regulates IVD analyzers, automation tracks, and associated software	Classifies devices (Class I–III); reviews 510(k), de novo, PMA; assigns CLIA complexity after clearance; regulates pre-analytical systems
Accreditation Bodies (e.g., CAP)	Provides accreditation beyond CLIA minimums	Evaluates on-site validation, workflow documentation, precision/accuracy verification, LIS integration, QC/QA programs
Manufacturers	Design, validate, and submit automation devices	Must supply performance data and intended-use claims; support labs in CLIA verification and installation validation

**Table 3:** **CLIA and FDA topics that laboratories must address when deploying automation and robotics**.

Topic	Practical Questions for Laboratories
Pre-analytical Quality (CLIA Subpart K)	How will robots affect specimen ID, labeling, and integrity? How will delays, temperature exposure, and routing failures be documented and corrected?
Device Classification & 510(k) Status	Is the device FDA-cleared? Does the planned use match the labeling? Is method comparison needed for the transition from manual to automated handling?
CLIA Complexity & IQCP	Do robots change workflow risk? Have IQCP and risk assessments been updated to include robotic failure modes?
LIS/Middleware Interfaces & Data Integrity	How are robot events mapped to LIS? What safeguards ensure traceability during handoffs between TLA, cobots, and AMRs?
Human–robot Safety & Inspection Readiness	Are safety policies documented? Are speed/force limits set? Can surveyors watch a robot run and access logs, incident reports, and QC data?

## Data Availability

No new data were created or analyzed in this study.

## Abbreviations

The following abbreviations are used in this manuscript:

AMR	Autonomous Mobile Robot
CLIA	Clinical Laboratory Improvement Amendments
IVD	In vitro diagnostics
TAT	Turn Around Time
TLA	Total Lab Automation
FDA	Food and Drug Administration
MiR	Mobile Industrial Robots
CAP	College of American Pathologists
LC-MS	Liquid Chromatography–Mass Spectrometry
LIS	Laboratory Information System
FTE	Full-Time Employee
